# Auditory evoked potential wave VI as an objective indicator of sedation depth in neonates undergoing chloral hydrate sedation: a double-blind randomized controlled study

**DOI:** 10.3389/fped.2025.1629088

**Published:** 2025-08-12

**Authors:** Zong Zheng, Shanpu Yang, Hongyan Liu, Zhimin Sheng

**Affiliations:** ^1^ Department of Neonatology, People’s Hospital of Yuhuan City, Taizhou, China; ^2^ Department of Anesthesiology, Wenling Maternity and Child Health Care Hospital, Taizhou, China

**Keywords:** chloral hydrate, neonatal sedation, auditory evoked potentials, wave VI, Ramsay scale

## Abstract

**Background:**

Neonatal sedation depth monitoring is critical yet depends on the subjective Ramsay scale when used and lacks objective biomarkers. Although auditory evoked potential (AEP) wave VI disappearance is linked to reduced consciousness, its use for neonatal sedation monitoring remains underexplored. We aimed to determine whether wave VI could function as an objective indicator of sedation levels in neonates.

**Methods:**

This prospective, double-blind, randomized trial enrolled 100 neonates requiring hearing screening. Participants were randomly assigned in a 4:1 ratio to either the treatment group (*n* = 80; 50 mg/kg oral chloral hydrate) or the control group (*n* = 20; 0.9% saline placebo). The treatment group was further divided into three subgroups according to Ramsay sedation scores, namely, level 4 (*n* = 22), level 5 (*n* = 23), and level 6 (*n* = 35), while the control group was divided into level 3 (*n* = 5), level 4 (*n* = 12), and level 5 (*n* = 3). All neonates received a standardized AEP test performed by an experienced audiologist. Sedation depth was evaluated using the Ramsay scale, and the latency and disappearance rate of wave VI were recorded and correlated with sedation levels. The receiver operating characteristic (ROC) curve was used to evaluate the predictive ability of wave VI latency in deep sedation, analyzing its sensitivity, specificity, and predictive values.

**Results:**

In the treatment group, wave VI disappearance rates increased in a sedation-dependent manner across the Ramsay Sedation Scale: 0% at level 4, 26% at level 5, and 68.6% at level 6 (*p* < 0.05). No wave VI disappearance was observed in the control group. ROC analysis demonstrated that wave VI latency predicted deep sedation (Ramsay ≥ 5) with an area under the curve of 0.861 (95% confidence interval: 0.746–0.975). The optimal latency cutoff was 8.465 ms (72.7% sensitivity, 86.2% specificity).

**Conclusion:**

AEP wave VI latency and disappearance are objective, sensitive, and specific indicators of sedation depth in neonates. With further validation, wave VI has the potential to become a reliable neurophysiological tool for precise sedation monitoring in neonates.

**Clinical Trial Registration:**

https://www.chictr.org.cn/index.html, identifier ChiCTR2300068407.

## 1 Introduction

Brainstem auditory evoked potential (BAEP) is a widely used and objective electrophysiological method for assessing hearing in neonates. Its recording involves capturing a series of rapid, sinusoidal waves that occur within 10–15 ms of a click stimulus delivered via earphones. This set is generally composed of seven stable waves, namely I–VII, among which wave V has the highest amplitude and is the last to disappear as the stimulus intensity decreases. Consequently, wave V has the highest discrimination rate (1). Auditory evoked potential (AEP) wave VI is the first reproducible sine wave detected after wave V (2). The precise neural origin of wave VI remains uncertain. Some studies suggest it may arise from the internal capsule (3), while others propose the medial geniculate body of the thalamus as its likely generator (4). Balogh et al. observed AEP wave VI in 53 of 54 healthy subjects, demonstrating its prevalence in normal physiology, while its absence correlated with coma severity (5).

Chloral hydrate is a short-acting sedative–hypnotic frequently used in neonates undergoing BAEP testing. It acts via activation of the pH-dependent human proton-activated chloride channel, producing benzodiazepine-like effects (6). The Ramsay Sedation Scale is a common evaluation method for the sedative effect of chloral hydrate, classifying it as mild (Ramsay score of 1–2), moderate (3–4), or deep (5–6) (7). For non-invasive testing procedures, a Ramsay score of at least 4 is typically required to ensure minimal movement and cooperation (7, 8).

In our clinical practice, we observed the absence of wave VI in AEP recordings from neonates under partial chloral hydrate sedation. This observation led us to hypothesize that wave VI disappearance may be associated with sedation depth. Therefore, we conducted a prospective controlled trial to investigate whether AEP wave VI latency and disappearance correlate with varying levels of sedation and to evaluate their potential as objective neurophysiological indicators of sedation depth in neonates.

## 2 Materials and methods

### 2.1 Design and study subjects

This prospective, controlled, double-blind study was approved by the Institutional Clinical Research Ethical Review Boards of Wenling Maternity and Child Health Care Hospital, Taizhou, China (No. 2023-IRB-005), and the People's Hospital of Yuhuan City, Taizhou, China [No. 2023(001)]. Written informed consent was obtained from the parents or legal guardians of all the enrolled neonates. The study was prospectively registered in the Chinese Clinical Trial Registry (ChiCTR2300068407) on 17 February 2023. We confirm that our study complied with the Consolidated Standards of Reporting Trials (CONSORT) guidelines.

The following inclusion criteria were used: gestational age ≥36 weeks, birth weight >2,000 g, singleton birth, and serum bilirubin levels within the first postnatal week below the 95th percentile on Bhutani's nomogram. Additionally, neonates who received their first automated auditory brainstem response and auditory brainstem response screening between postnatal days 7 and 14 (between 20 March and 20 December 2023) were enrolled. The following exclusion criteria was applied: (1) multiple births; (2) severe asphyxia requiring resuscitation (Apgar score of 0–4 at 1 min or 0–6 at 5 min); (3) positive screening for neonatal genetic metabolic diseases or glucose-6-phosphate dehydrogenase deficiency; (4) hypoxemia or mechanical ventilation >5 days; (5) family history of permanent childhood hearing impairment or congenital infections (cytomegalovirus, rubella, herpes, or syphilis); (6) craniofacial deformities involving the auricle or ear canal; (7) prenatal or postnatal exposure to known ototoxic medications (such as aminoglycosides and loop diuretics) or maternal use of ototoxic drugs during pregnancy; (8) bacterial meningitis; (9) syndromes/genetic disorders associated with hearing impairment; (10) serum bilirubin >342 μmol/L within the first 7 days; (11) incomplete or unreliable data, or parental refusal; (12) contraindications to chloral hydrate (such as allergy, severe hepatic/renal/cardiac dysfunction, and/or porphyria).

### 2.2 Study protocol

Randomization was performed by an independent statistician using computer-generated block randomization codes generated using MedCalc for Windows (version 18.2.1, Ostend, Belgium). Allocation codes were sealed in sequentially numbered, individual, opaque envelopes to ensure concealment. A research nurse not involved in clinical care prepared identical 10 mL syringes labeled “study drug,” each containing either 0.5 mL/kg of 10% chloral hydrate (50 mg/kg) or an equivalent volume of saline placebo, according to the randomized code in the envelope. The prepared syringes were then delivered to the anesthesiologists who, along with the audiologist, remained blinded to the group assignments.

Prior to AEP testing, neonates were screened to exclude external auditory canal obstruction, middle-ear effusion, agitation (restlessness and/or crying), excessive ambient noise, and other confounders. Parents or legal guardians received information about the procedure verbally, including details about the intervention and the potential risks, and provided written informed consent. A brief medical history and physical examination were then performed, and the documented parameters included level of consciousness, baseline physiological measures (Masimo Rad-57 pulse oximeter; Neonatology Care Group, California, USA; batch N115622), and demographic data.

Each neonate received the assigned oral solution—chloral hydrate (50 mg/kg) or saline placebo (0.5 mL/kg)—while seated on their legal guardian's lap in a quiet room. Once the neonate fell asleep spontaneously and did not wake up despite mild skin stimulation, they were transferred to the testing room equipped with comprehensive resuscitation facilities. Sedation depth and vital signs were monitored every 5 min. If a Ramsay score of ≥4 was not achieved within 30 min, a supplemental half-dose was administered. Sedation failure was documented if the Ramsay score remained <4 1 hour after the second dose.

The sedative effect of chloral hydrate was evaluated using an adapted Ramsay scoring method based on observable behavioral responses, as follows: level 1: restless and agitated, with inability to settle; level 2: quiet yet still somewhat active; level 3: calm and drowsy, exhibiting minimal spontaneous movement; level 4: asleep but responsive to strong external stimuli (such as a loud noise or gentle touch); level 5: asleep with only minimal response to strong stimuli; level 6: deeply asleep without response to stimuli of any intensity (7). A Ramsay score of  ≥ 4 was considered to indicate effective sedation, and a minimum sedation level of 4 was required to perform the standardized AEP test (7, 8).

AEPs were recorded using the ICS Chartr EP200 auditory evoked potential testing system. Testing parameters adhered to the ICS Chartr EP 200 Operation Guide. The impedances of all four electrodes were maintained below 5 kohms, with a maximum difference of 2 kohms between the left and right electrodes. The stimulus rate was set to 21.1/s, and responses were averaged over 1,024 scans. Left-ear stimulation commenced at 60 dBnHL. Once a repeatable sine wave was observed within the expected latency window (approximately 8.0 ms after wave V), it was recorded as wave VI. If no repeatable wave was detected, the intensity was increased by 10 dBnHL increments, up to 80 dBnHL. The corresponding intensity and latency were recorded if a wave VI was elicited; otherwise, “no AEP wave VI” was recorded. Following completion of the test, the electrodes were removed, and peripheral blood oxygen saturation, heart rate, and blood pressure were continuously monitored using non-invasive oscillometric devices. This monitoring spanned from sedation until full awakening, and all neonates remained under observation in the recovery room for at least 24 h. Neonates who failed to arouse after the test were further monitored by neonatologists until their vital signs and consciousness returned to the pre-test state. Upon return to pre-procedure health status, neonates were discharged in accordance with the American Society of Anesthesiologists’ guidelines.

### 2.3 Data collection and outcome assessment

The primary outcome was the disappearance rate of AEP wave VI (defined as the absence of a reproducible waveform at up to 80 dBnHL) and wave VI latency and their predictive value for deep sedation (Ramsay score of ≥5).

Secondary outcomes included the incidence of prolonged latency and the incidence of unilateral and bilateral wave VI disappearance at different sedation levels.

### 2.4 Sample size estimation

The sample size was determined using PASS 2021 software (NCSS, Kaysville, UT, USA) based on the primary outcome of wave VI disappearance rate in the treatment group. A previous pilot study indicated that the wave VI disappearance rate under deep sedation (Ramsay score of ≥5) was approximately 40%, while no disappearance was observed in the placebo group (0%). To detect this difference with a two-sided *α* of 0.05, power of 80%, and an allocation ratio of 4:1 (treatment:control), a minimum of 72 neonates in the treatment group and 18 neonates in the control group was required. Considering potential attrition rates estimated at 10% from prior neonatal sedation trials, we increased the sample size to 80 neonates in the treatment group and 20 in the placebo group, resulting in a total of 100 participants.

### 2.5 Statistical analysis

All analyses were conducted using SPSS 22.0 (IBM, Armonk, NY, USA). Continuous variables were first tested for normality using the Shapiro–Wilk test. Normally distributed data are reported as mean ± SD and compared using one-way ANOVA with Bonferroni *post hoc* correction across Ramsay subgroups. Non-normal data are presented as median (interquartile range) and compared using the Kruskal–Wallis test. Categorical variables are expressed as n (%) and compared using the *χ*^2^ test or Fisher's exact test, as appropriate. Trends in wave VI disappearance rates across increasing Ramsay levels were evaluated by the Cochran–Armitage trend test. Receiver operating characteristic (ROC) curves—constructed to assess the ability of wave VI latency to predict deep sedation (Ramsay ≥5)—and the area under the curve (AUC) with 95% CI, optimal cut-off (Youden index), sensitivity, and specificity, are reported. All tests were two-tailed, and *P* < 0.05 was considered statistically significant.

## 3 Results

**Figure 1:**
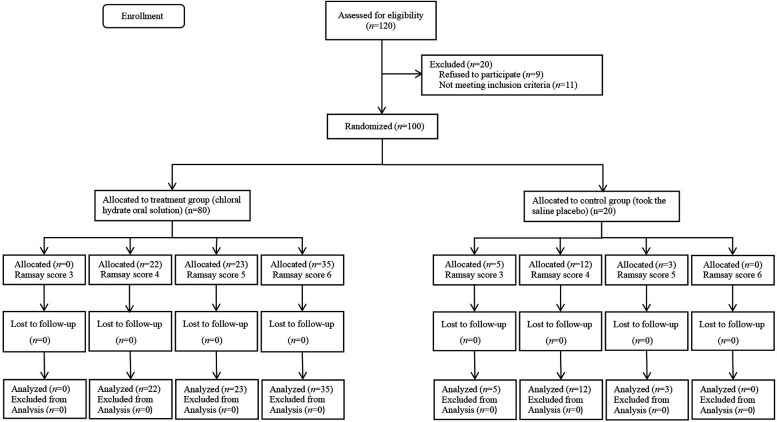
CONSORT diagram showing neonate recruitment and the study workflow.

A total of 120 neonates were recruited and assessed in this study, with 20 excluded (11 failed to meet the inclusion criteria and 9 refused to participate); thus, 100 neonates were enrolled in the final analysis. The study flowchart is shown in Figure 1. There was no significant difference between the 80 cases in the treatment group and the 20 cases in the control group in terms of phototherapy, gender, gestational age, birth mode, birth weight, and proportion of small for gestational age (SGA) (*P* > 0.05). In the treatment group, there were 30 cases of wave VI disappearance, and the difference was statistically significant compared with the control group (*P* < 0.01) (Table 1).

**Table 1:** Comparison of basic information and wave VI disappearance cases between groups.

Variables	Treatment group (*n* = 80)	Control group (*n* = 20)	*P*-value
Male	37 (46)	10 (50)	0.764
Premature baby	5 (6)	3 (15)	0.197
Low birth weight infants (<2,500 g)	5 (6)	3 (15)	0.197
Small for gestational age	6 (8)	2 (10)	0.712
Used NCPAP	3 (4)	2 (10)	0.251
Caesarean section	34 (43)	10 (50)	0.546
Premature rupture of membranes at >18 h	5 (6)	3 (15)	0.197
Invasive mechanical ventilation	1 (1)	1 (5)	0.264
TSH > 20 mIU/L	3 (4)	2 (10)	0.251
Phototherapy	38 (48)	13 (65)	0.161
Wave VI disappearance cases	30 (38)	0 (0)	<0.01

Data are presented as the number (%).

NCPAP, nasal continuous positive airway pressure; TSH, thyroid-stimulating hormone.

**Figure 2:**
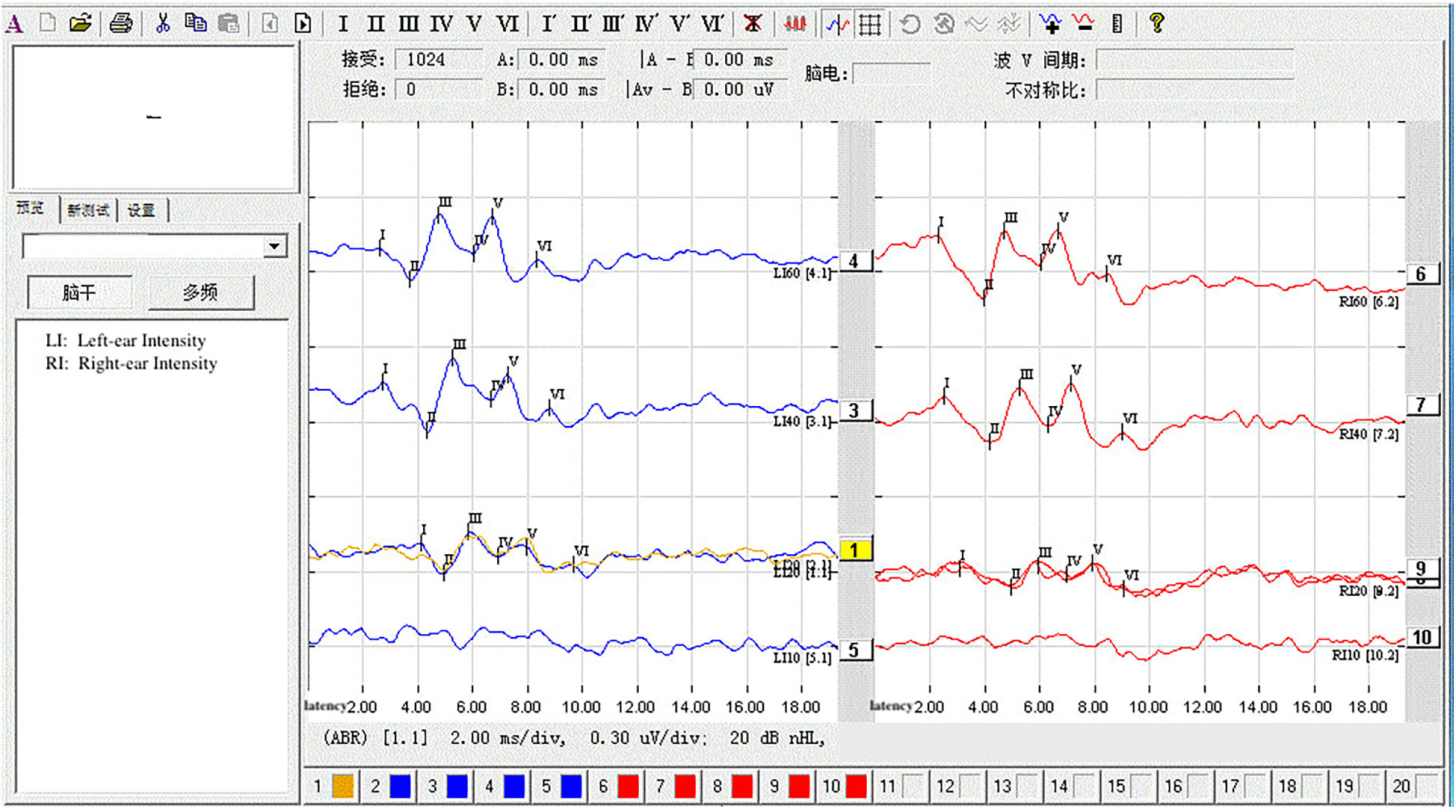
AEP recording showing wave VI under normal physiological conditions.

**Figure 3:**
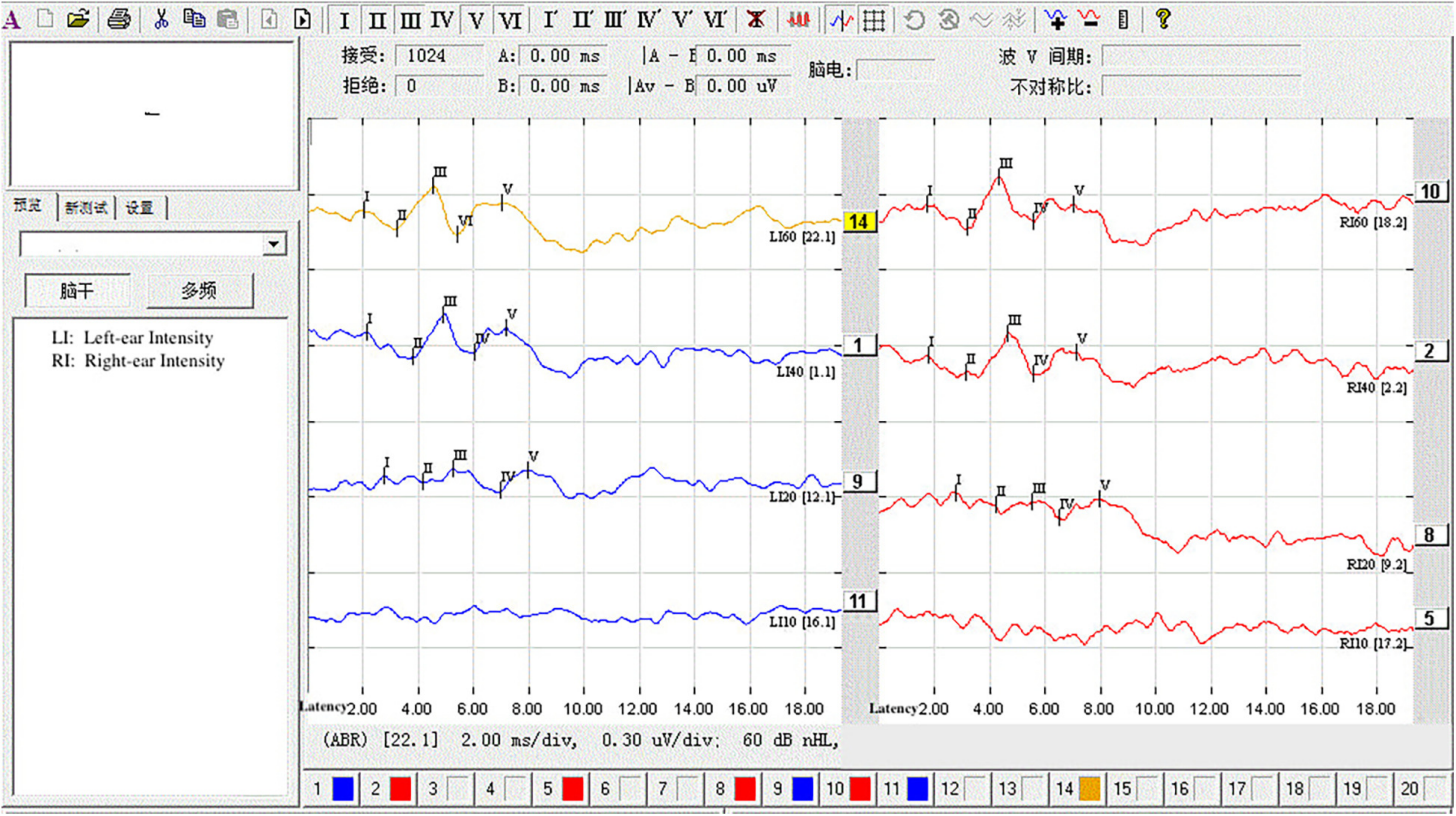
Under deep sedation (Ramsay score of 6), AEP at 60 dB failed to elicit wave VI. The stimulus intensity was increased in 10 dB steps up to 80 dB, but wave VI remained undetectable.

**Figure 4:**
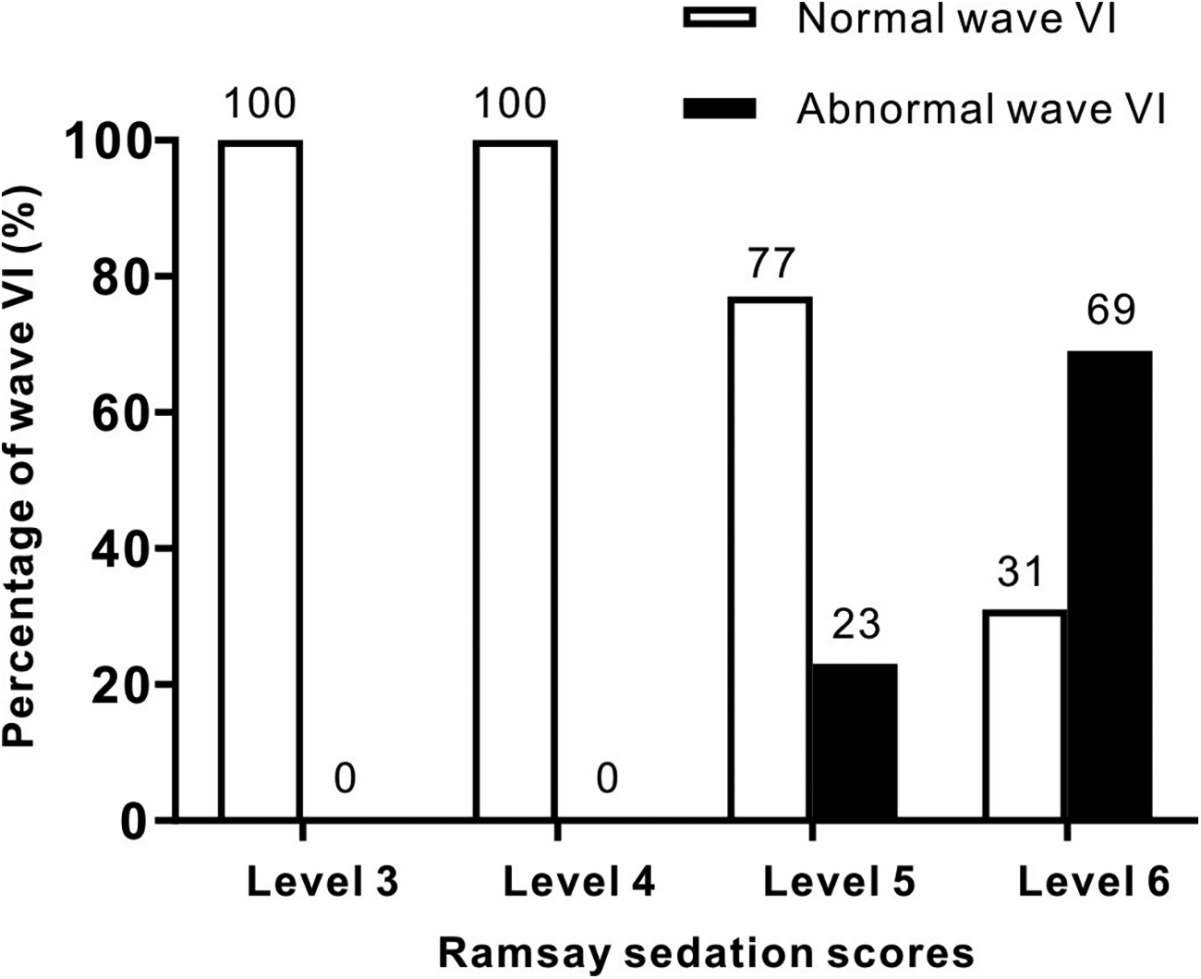
Trend correlation between AEP wave VI outcomes and Ramsay sedation scores in the treatment and control groups. Bars represent the number of normal (white) and abnormal (black) wave VI detections. A significant trend was found using the Cochran-Armitage test (*P* < 0.01).

The statistical analysis of the wave VI results from the neonates in the treatment group revealed that of the 35 neonates with a Ramsay score of 6, 14 cases had unilateral disappearance of AEP wave VI, and 10 cases had bilateral wave VI disappearance. The proportion of positive cases was 68.6%. Among the 23 neonates with a Ramsay score of 5, AEP wave VI disappeared unilaterally in four cases and bilaterally in two cases, accounting for 26.0% of the positive cases. The frequency of bilateral or unilateral wave VI disappearance increased significantly with an increase in the Ramsay score (*P* < 0.05) (Table 2). In the control group, no disappearance of wave VI was found (Table 3). Representative AEP tracings are presented in Figure 2 (wave VI under normal physiological conditions) and Figure 3 (absence of wave VI despite maximal stimulation intensity of 80 dBnHL under deep sedation). The AEP thresholds (wave V detection thresholds) did not differ significantly between the treatment and control groups: for the left ear, the treatment group averaged 22.1 ± 4.9 dBnHL vs. 23.0 ± 6.2 dBnHL in controls (*p* = 0.473); for the right ear, the values were 23.9 ± 5.6 dBnHL vs. 25.5 ± 5.6 dBnHL, respectively (*p* = 0.250) (Table 4). The Cochran–Armitage analysis revealed a significant relationship between Ramsay score and wave VI outcomes (*P* < 0.01) (Figure 4).

**Table 2:** Synopsis of findings in the treatment group.

Variable	Ramsay score of 3	Ramsay score of 4	Ramsay score of 5	Ramsay score of 6	*P*-value
Number of neonates	0	22	23	35	
Unilateral disappearance of wave VI	0	0	4	14	0.002
Bilateral disappearance of wave VI	0	0	2	10	0.008
Unilateral prolonged latency of wave VI	0	1	3	18	<0.01
Bilateral prolonged latency period of wave VI	0	0	2	9	0.016

Data are presented as numbers.

**Table 3:** Synopsis of findings in the control group.

Variable	Ramsay score of 3	Ramsay score of 4	Ramsay score of 5	Ramsay score of 6	*P*-value
Number of neonates	5	12	3	0	
Unilateral disappearance of wave VI	0	0	0	0	–
Bilateral disappearance of wave VI	0	0	0	0	–
Unilateral prolonged latency of wave VI	0	0	1	0	0.150
Bilateral prolonged latency period of wave VI	0	0	0	0	–

Data are presented as numbers.

**Table 4:** Comparison of AEP thresholds between groups.

AEP threshold	Treatment group (*n* = 80)	Control group (*n* = 20)	*P*-value
Left-ear threshold	22.1 ± 4.9	23.0 ± 6.2	0.473
Right-ear threshold	23.9 ± 5.6	25.5 ± 5.6	0.250

Data are presented as mean ± SD dBnHL.

**Figure 5:**
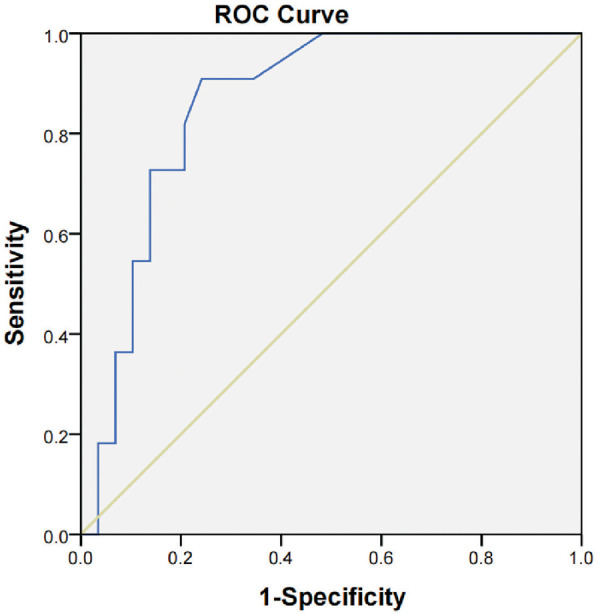
ROC curve showing the predictive value of AEP wave VI latency for AEP wave VI disappearance under deep sedation. The AUC was 0.861 (95% CI: 0.746–0.975, *P* < 0.001). At a Youden index of 0.589, the optimal latency cutoff was 8.465 ms, with a sensitivity of 72.7%, a specificity of 86.2%, a negative predictive value (NPV) of 89.2%, and a positive predictive value (PPV) of 76.2%.

The ROC analysis demonstrated that the latency of wave VI is a significant predictor of its disappearance under deep sedation (Ramsay score > 5), with an AUC of 0.861 [95% confidence interval (CI): 0.746–0.975, *P* < 0.001]. At a Youden index of 0.589, the optimal cut-off latency was 8.465 ms, resulting in a sensitivity of 72.7%, a specificity of 86.2%, a negative predictive value of 89.2%, and a positive predictive value of 76.2% (Figure 5).

## 4 Discussion

Chloral hydrate is the most commonly used sedative for routine neonatal examinations due to its efficacy and safety (9). Its mechanism of action parallels that of benzodiazepines (6). It provides rapid onset, deep sedation, prolonged duration, and rapid recovery, making it particularly suitable for neonatal AEP testing (10). With dosing protocols guided by experienced anesthesiologists over decades of neonatal clinical practice, its use has become well-established (11). However, individual variability persists, primarily due to factors including body weight, gestational age, and postnatal age. Despite extensive clinical experience, precise control of sedation depth remains challenging, as excessive sedation may induce life-threatening complications, including arrhythmia, respiratory depression, and airway obstruction (12). Therefore, an objective and effective indicator is urgently needed to accurately assess sedation depth.

The Ramsay scoring method is a simple and rapid tool for assessing sedation level and is supported by extensive clinical experience (13). Scoring requires an experienced clinician to evaluate behavioral responses at 10-minute intervals. This approach offers moderate accuracy but lacks objectivity. Consequently, research efforts have focused on identifying physiological indicators with greater sensitivity and specificity for sedation monitoring.

The clinical application of BAEP began in the early 1970s (14). Clinical research has demonstrated that AEP abnormalities in comatose patients with traumatic brain injuries manifest as the disappearance of wave VI (5). This phenomenon is thought to result from inhibition of the medial geniculate body (MGB) in the thalamus (15). The relay nucleus of the auditory radiation—composed of fibers from the primary auditory nucleus—transmits auditory signals through the posterior limb of the internal capsule to the auditory cortex, serving as a secondary processing center in the auditory pathway. Under deep sedation, inhibition of the thalamic MGB may disrupt signal transmission along the auditory pathway, reducing the information processing load on the cerebral cortex and thereby decreasing overall neural activity (16). Because the MGB within the internal capsule receives but does not relay feedback signals under inhibition, the corresponding AEP wave VI fails to appear (17). However, the precise neural mechanism underlying wave VI disappearance requires further investigation.

Owing to its non-invasive nature, objectivity, and high sensitivity, BAEP measurements remain stable across varied external conditions. Consequently, it has been widely applied to assess hearing and central nervous system dysfunction in neonatology, such as perinatal asphyxia, cerebellar atrophy, intrauterine growth restriction, encephalitis, bilirubin encephalopathy, and intracranial hemorrhage (18). AEP waveforms comprise seven peaks (waves I–VII), each originating from distinct neural generators. Wave I reflects auditory nerve action potentials, wave II arises from the cochlear nucleus, wave III from the pontine olivary complex, and waves IV and V correspond to activity in the lateral lemniscus and inferior colliculus, respectively. Waves VI and VII originate from the medial geniculate body and thalamic auditory radiations, respectively. The latencies of these seven waves index conduction times along successive segments of the auditory pathway (19). Prolonged latency of any wave indicates abnormalities in its corresponding conduction pathway, thus serving as an indicator of dysfunction (20). Therefore, clinical investigations of wave VI abnormalities should include latency analysis. Prolonged wave VI latency, wave VI disappearance, and reduced wave VI amplitude constitute positive indicators of subclinical auditory pathway suppression. As inhibitory neuronal activity increases, the wave VI signal weakens progressively, with latency increasing until complete disappearance. This progression is observed in brainstem evoked potentials during the gradual onset of chloral hydrate effects after neonatal oral administration.

Using the Ramsay Sedation Scale as the reference standard, we generated ROC curves of wave VI latency to predict its disappearance under deep sedation. The ROC analysis yielded an AUC of 0.861, with an optimal latency cutoff at 8.465 ms, corresponding to 72.7% sensitivity, 86.2% specificity, 89.2% negative predictive value, and 76.2% positive predictive value. These results indicate that prolonged wave VI latency may correlate with its eventual disappearance during chloral hydrate-induced sedation. Notably, recent research by Claesdotter-Knutsso et al. investigating the influence of methylphenidate on auditory evoked responses in patients with attention deficit hyperactivity disorder has also reported medication-induced alterations in wave VI (21). While their study involved a different patient population and medication, it offers additional evidence that pharmacological agents can alter BAEP waveforms. However, because our study focused solely on the effects of chloral hydrate, the applicability of these findings is limited to sedation induced by this agent. Incorporating a positive control group using alternative sedatives would strengthen generalizability. Future research should therefore investigate whether wave VI presence and latency reliably reflect varying depths of consciousness or sedation across different pharmacological agents and clinical settings.

Our study had certain limitations. First, subjects were stratified by Ramsay score to form three groups with progressively increasing sedation levels. Based on the abnormal wave VI incidence per group, we found a significant increase in wave VI loss frequency with higher Ramsay scores (*P* < 0.05). This suggests a positive correlation between sedation depth and wave VI alterations; confirming whether this relationship is linear requires validation in future studies with larger sample sizes. Second, none of the 20 control group cases exhibited wave VI disappearance during wakefulness or light sleep. This implies that drug-induced sedation may be a prerequisite for using wave VI disappearance as a sedation depth indicator, which requires further verification. Third, we found that wave VI disappearance incidence increased with sedation depth (Ramsay score of ≥5), reaching approximately 66.7% under deep sedation. The single-ear incidence of wave VI disappearance was slightly higher than that from two ears, but whether there is a relationship between the two is still unclear based on the current data. Future studies with larger samples should investigate whether there are statistical differences in left/right ear wave VI abnormalities.

## 5 Conclusion

In summary, AEP wave VI disappearance and latency serve as objective indicators of sedation depth during chloral hydrate-induced anesthesia in neonates, exhibiting high specificity and sensitivity. This holds significant importance for accurate sedation assessment and precision anesthesia practice. With further research, wave VI may emerge as a reliable indicator for sedation assessment, while its latency could serve as a potentially valuable metric for anesthesia evaluation.

## Data Availability

The original contributions presented in the study are included in the article/Supplementary Material, further inquiries can be directed to the corresponding author.
